# Pharmacists as important prescribers of coronavirus disease 2019 (COVID-19) antivirals

**DOI:** 10.1017/ash.2022.248

**Published:** 2022-07-11

**Authors:** Monica V. Mahoney, Hita Bhagat, Robbie Christian, Carlos del Rio, Kenneth C. Hohmeier, Michael E. Klepser, Jason M. Pogue

**Affiliations:** 1 Department of Pharmacy, Beth Israel Deaconess Medical Center, Boston, Massachusetts; 2 Department of Pharmacy, Community Health Network, Indianapolis, Indiana; 3 Baton Rouge General, Baton Rouge, Louisiana; 4 Division of Infectious Diseases, Department of Internal Medicine, Emory University School of Medicine, Atlanta, Georgia; 5 Department of Clinical Pharmacy & Translational Science, University of Tennessee Health Science Center, Nashville, Tennessee; 6 Ferris State University College of Pharmacy, Big Rapids, Michigan; 7 Department of Clinical Pharmacy, University of Michigan College of Pharmacy, Ann Arbor, Michigan

## Abstract

Although pharmacists are key members of the healthcare team, they are currently ineligible to independently prescribe the oral coronavirus disease 2019 (COVID-19) antivirals. We report the roles pharmacists have undertaken during the COVID-19 pandemic and provide evidence for the support of independent oral COVID-19 antiviral prescribing.

Pharmacists play a vital role in healthcare and public health. With 91% of Americans living within 5 miles of a community pharmacy^1^ and with longer business hours than traditional medical offices, pharmacies are commonly used as care access points. Medicare beneficiaries visit community pharmacies an average of 6–7 additional times annually compared to primary care physicians (PCPs), with the largest discrepancies in rural areas.^2^ Not only are pharmacists highly accessible healthcare providers, they are also consistently viewed as one of the most trustworthy professions.^3^

This accessibility and trustworthiness have been paramount during the coronavirus disease 2019 (COVID-19) pandemic. Pharmacists have been essential in expanding vaccination efforts, especially in underserved communities. Of the 559 million US COVID-19 vaccine doses administered, >234 million (42%) were administered in pharmacies.^4^ Given discrepancies in national vaccination rates with lower uptake in rural^5^ and minoritized communities,^6,7^ pharmacists have proven instrumental in decreasing inequities through innovative practices and greater accessibility.^7^

Sadly, in the United States, inequities related to social determinants of health in COVID-19 pervade beyond vaccination efforts. Although severe acute respiratory coronavirus virus 2 (SARS-CoV-2) infection rates in rural areas have been similar to those in urban areas, death rates are 33% higher.^8^ Non-Hispanic Black, Hispanic/Latino, and Native American persons are twice as likely to be hospitalized or die from COVID-19 than White persons.^9^ Excess mortality vulnerability factors in rural areas include older age, increased comorbid conditions, lack of health insurance, and distance to hospitals with intensive care units.^10^

The authorization of monoclonal antibodies was a groundbreaking advance, offering our first therapeutic option that could significantly decrease the risk of hospitalization or death.^11–13^ Unfortunately, accessibility inequities with these agents have also been evident over the past 18 months. In addition to medication shortages, the monoclonal antibodies are often not used in the highest-risk patients. Reasons for this include difficulty navigating the healthcare system, limited and complicated treatment availability, time constraints between diagnosis and administration, and geographic limitations.^14^ Additionally, Black, Asian, and other non-White races received monoclonal antibody therapy 22%, 48%, and 47% less frequently than their White counterparts, respectively.^15^ Pharmacists have been instrumental in increasing access to monoclonal antibodies in rural and minoritized communities,^16^ subsequently increasing the number of Black patients receiving treatment.^17^

In December 2021, the first effective oral antiviral agents were authorized by the FDA for high-risk patients in the United States.^18–21^ Although these agents have the potential to broaden access to early treatment and to reduce inequities given the ease of administration, there are significant barriers to success. These include the need for rapid initiation within 5 days of symptom onset, the requirement for a positive test result, and several pharmacologic considerations requiring careful prescribing (ie, drug interactions, renal dosing, and teratogenicity). Recognizing these barriers, President Biden launched a nationwide test-to-treat initiative.^22^ Pillars of this initiative include pharmacy-based community clinics offering on-the-spot SARS-CoV-2 testing and immediate access to treatment for qualifying patients. Unfortunately, the small percentage of community pharmacy chain locations with prescriber-based clinics (only 11% of CVS pharmacies^23^ and 4% of Walgreens pharmacies^24^), the absence of such clinics at independent pharmacies, and the limited hours of federally qualified community health centers will unnecessarily impede the ability to access these facilities and will further worsen inequities.

Pharmacists are well positioned to navigate many of these barriers and have documented experience improving outcomes and access with test-to-treat initiatives, including respiratory infections. Although the Ninth Amendment of the PREP Act authorized pharmacists to “order and administer select COVID-19 therapeutics” that are “administered subcutaneously, intramuscularly, or orally in accordance with the FDA approval, authorization, clearance, or licensing,”^25^ the language within the emergency use authorization (EUA) for both oral agents specifically states that they “may only be prescribed for an individual patient by physicians, advanced practice registered nurses, and physician assistants.”^18,19^ As a result, these EUAs override the Ninth Amendment of the PREP Act, prohibit pharmacists from prescribing the oral antivirals independently, and severely compromise the test-to-treat initiative.

Community pharmacies have ideal blueprints and footprints for independently prescribing oral anti–COVID-19 therapeutics. Additionally, community pharmacists have close relationships with the communities they serve. This familiarity allows for improved education, awareness, and advocacy of therapeutics to patients experiencing symptoms of COVID-19. Since early in the pandemic, community pharmacists have been at the forefront of COVID-19 testing. Because prompt initiation of treatment (within 5 days) or onset of symptoms is required for these drugs to be effective, testing followed by prompt initiation of therapy is critical. Of the 20,000 federally supported, free testing sites nationwide, community pharmacies comprise at least half of the sites.^26^ Because patients are used to going to the pharmacy for SARS-CoV-2 testing, a natural extension would be to allow pharmacists the authority to independently prescribe an oral anti–COVID-19 therapeutic to eligible patients who test positive. This reduces barriers for patients who qualify for therapies, decreases the time to treatment, and limits exposures to infectious individuals.

Similar collaborative community pharmacy-based models for disease management have proven safe and effective for other acute infectious conditions such as group A *Streptococcus*, influenza, and HIV pre-exposure prophylaxis.^27–29^ These models are used in several countries including the United States, Canada, England, and New Zealand. Relating to pandemic inequities, data show that upward of 54% of individuals using these services report not having a primary care physician and 38% presented outside traditional clinic hours for evaluation.^27^ Algorithms incorporating risk factors and clinical presentations direct pharmacists to refer patients for higher-level care (eg, emergency department, urgent care, or primary care physician) when necessary, versus determining which patients require anti-infectives or over-the-counter symptomatic relief.^28^ In fact, these models were initially created to develop infrastructure and skills to allow pharmacies to offer a higher degree of care should a pandemic arise. As result of the experiences with influenza and acute pharyngitis care models, a similar service can be developed for the COVID-19 pandemic. Because screening and testing for SARS-CoV-2 already occur in pharmacies, all that is needed is a collaborative practice agreement (CPA) allowing an oral antiviral to be dispensed. A CPA could easily be developed using the existing parameters for use that are included within the EUA for each of the medications. During times of crisis, the full criteria for use could be incorporated into a template CPA and endorsed by the FDA.

Although significant treatment nuances exist regarding use of currently available COVID-19 oral antivirals, these considerations make a compelling argument for pharmacist prescribing rather than against it. Pharmacists are drug experts. Their education on pharmacology, agent selection, dosing, adverse effects, and drug interactions is vastly more extensive than other healthcare professionals. Drug interactions are a major concern with nirmatrelvir/ritonavir, and notably the National Institutes of Health guidelines highlight that clinicians should consult an expert, such as a pharmacist, prior to use.^30^ Although COVID-19–specific drug-interaction websites exist, access to an accurate medication record, knowledge of how to interpret or adjust concomitant medications, and ability to counsel patients on managing interactions is imperative to prescribing nirmatrelvir-ritonavir. In addition to their drug expertise and experience managing drug interactions, pharmacists are well versed in taking medication histories, and many times the pharmacy is the only place where an accurate medication list exists. This combination makes pharmacists ideally positioned to independently prescribe nirmatrelvir-ritonavir for most patients. When less straightforward and more complex patient scenarios occur, pharmacists can initiate additional discussion with a patient’s primary care physician or specialist and can work collaboratively to determine the optimal course of action. Importantly, this is no different than what should occur in these scenarios if another EUA-approved healthcare provider currently prescribed nirmatrelvir-ritonavir at a pharmacy-based clinic.

Additional pharmacological concerns include renal dosage adjustment with nirmatrelvir-ritonavir^18^ and teratogenicity concerns with molnupiravir.^19^ Pharmacists are well positioned and experienced to address these issues as well. With more complete access to medication records, pharmacists can identify other renally adjusted medications or medications prescribed for complications of kidney disease. Community pharmacists have demonstrated the ability to accurately identify patients who require renal dosing adjustments comparable to prescribers.^31^ Alternatively, under a CPA, pharmacists can be authorized to order laboratory tests for patients to assess for appropriateness of drug therapies. Furthermore, pharmacists routinely counsel patients regarding teratogenicity risks and the necessary precautions with such therapies.

Supplemental training for pharmacists is required for appropriate diagnostic utilization, interpretation, and medication prescribing. Several national pharmacy organizations have readily available training programs. The National Alliance of State Pharmacy Associations has a certificate training program for pharmacy-based point-of-care and test-to-treat for various conditions/pathogens including coronaviruses, influenza, group A streptococcus, HIV, and hepatitis C.^32^ This program provides updates on relevant disease states, reviews physical assessment and triaging skills, and provides examples of incorporating disease management services. To date, ∼7,000 pharmacists have completed this program. Further, according to the PREP Act requirements, training was necessary for pharmacists to prescribe and administer COVID-19 monoclonal antibodies, and organizations such as the Society of Infectious Diseases Pharmacists and the American Society of Health-System Pharmacists provide such resources.^33,34^ A similar approach could be applied to the oral antivirals. Notably, the Ninth Amendment of the PREP Act grants liability protection to pharmacists. If the PREP Act were to expire and pharmacists’ authority were granted under a CPA or protocol, liability would only be assumed if the pharmacist deviated from the CPA or protocol.

Most importantly however, community pharmacies need further resources before they can independently prescribe oral therapeutics. The EUA language needs to be amended, allowing pharmacists the authority to prescribe. Adequate reimbursement must be provided for the cognitive and dispensing services provided. This begins with legislation allowing pharmacists to bill Medicare Part B for services provided to its beneficiaries, such as the Pharmacy and Medically Underserved Areas Enhancement Act.^35,36^ Receiving Medicare B recognition and reimbursement will in turn allow for additional staffing models to absorb the increased work required and will allow time for appropriate patient care.

In conclusion, pharmacies represent an increasingly critical access point for disease prevention and treatment for millions of Americans. Pharmacists have proven themselves over several decades to be integral members of the healthcare team. They have a high degree of community trust, and they are too often the only access to healthcare in the United States. The availability of highly effective oral antivirals and a test-to-treat plan are critical steps in limiting morbidity and mortality due to COVID-19. However, its success is dependent on the ability to reach members of every community, particularly communities disproportionately devastated by the pandemic.^37,38^ Pharmacists have proven their ability to test to treat with outpatient respiratory infections, improving both access and outcomes. With appropriate training and financial compensation, pharmacists are optimally situated to collaboratively achieve this goal with other healthcare providers.

## References

[1] Qato DM , SZenk S , Wilder J , Harrington R , Gaskin D , Alexander GC . The availability of pharmacies in the United States: 2007–2015. PLoS ONE 2017;12:e0183172. 10.1371/journal.pone.0183172 PMC5559230 28813473

[2] Berenbrok LA , Gabriel N , Coley KC , Hernandez I. Evaluation of frequency of encounters with primary care physicians vs visits to community pharmacies among Medicare beneficiaries. JAMA Network Open 2020;3:e209132. 32667653 10.1001/jamanetworkopen.2020.9132 PMC7364370

[3] Gallup poll website. https://news.gallup.com/file/poll/388700/220112HonestyEthics.pdf. Accessed March 23, 2022.

[4] Federal retail pharmacy program. Centers for Disease Control and Prevention website. https://www.cdc.gov/vaccines/covid-19/retail-pharmacy-program/index.html. Accessed March 23, 2022.

[5] Saelee R , Zell E , Patel Murthy B , et al. Disparities in COVID-19 vaccination coverage between urban and rural counties—United States, December 14, 2020–January 31, 2022. Morb Mort Wkly Rep 2022;71:335–340. 10.15585/mmwr.mm7109a2 PMC8893338 35239636

[6] Marcelin JR , Swartz TH , Bernice F , et al. Addressing and inspiring vaccine confidence in black, indigenous, and people of color during the coronavirus disease 2019 pandemic. Open Forum Infect Dis 2021;8:ofab417. 34580644 10.1093/ofid/ofab417 PMC8385873

[7] Abdul-Mutakabbir JC , Caset S , Jews V , et al. A three-tiered approach to address barriers to COVID-19 vaccine delivery in the black community. Lancet Glob Health 2021;9:e749–e750. 33713634 10.1016/S2214-109X(21)00099-1 PMC7946412

[8] Ullrich F , Mueller K. COVID-19 cases and deaths, metropolitan and nonmetropolitan counties over time (update). RUPRI Center for Rural Health Policy Updates website. https://rupri.public-health.uiowa.edu/publications/policybriefs/2020/COVID%20Longitudinal%20Data.pdf. Accessed March 23, 2022.

[9] COVID-19 hospitalization and death by race/ethnicity. Centers for Disease Control and Prevention website. https://www.cdc.gov/coronavirus/2019-ncov/covid-data/investigations-discovery/hospitalization-death-by-race-ethnicity.html. Accessed April 2, 2022.

[10] Dobis EA , McGranahan D. Rural residents appear to be more vulnerable to serious infection or death from coronavirus COVID-19. US Department of Agriculture website. https://www.ers.usda.gov/amber-waves/2021/february/rural-residents-appear-to-be-more-vulnerable-to-serious-infection-or-death-from-coronavirus-covid-19. Accessed March 23, 2022.

[11] Gupta A , Conzalez-Rojas Y , Juarez E , et al. Effect of sotrovimab on hospitalization or death among high-risk patients with mild-to-moderate COVID-19: a randomized clinical trial. JAMA 2022;327:1236–1246. 35285853 10.1001/jama.2022.2832 PMC8922199

[12] Weinreich DM , Sivapalasingam S , Norton T , et al. REGEN-COV antibody combination and outcomes in outpatients with COVID-19. N Engl J Med 2021;385:e81. 34587383 10.1056/NEJMoa2108163 PMC8522800

[13] Gottlieb RL , Nirula A , Chen P , et al. Effect of bamlanivimab as monotherapy or in combination with etesevimab on viral load in patients with mild to moderate COVID-19: a randomized clinical trial. JAMA 2021;325:632–644. 33475701 10.1001/jama.2021.0202 PMC7821080

[14] Behr CL , Joynt Maddox KE , Meara E. Anti–SARS-CoV-2 monoclonal antibody distribution to high-risk medicare beneficiaries, 2020–2021. JAMA 2022;327:980–983. 35119452 10.1001/jama.2022.1243 PMC8904305

[15] Wiltz JL , Feehan AK , Molinary NAM , et al. Racial and ethnic disparities in receipt of medications for treatment of COVID-19—United States, March 2020–August 2021. Morbid Mortal Wkly Rep 2021;71:96–102. 10.15585/mmwr.mm7103e1 PMC8774154 35051133

[16] Bariola JR , McCreary EK , Wadas RJ , et al. Impact of bamlanivimab monocloncal antibody treatment on hospitalization and mortality among nonhospitalized adults with severe acute respiratory syndrome coronavirus 2 infection. Open Forum Infect Dis 2021;8:ofab254. 34250192 10.1093/ofid/ofab254 PMC8241472

[17] McCreary EK , Bariola JR , Minnier T , et al. A learning health system randomized trial of monoclonal antibodies for COVID-19. *medRxiv* 2021. doi: 10.1101/2021.09.03.21262551.

[18] Paxlovid emergency use authorization. US Food and Drug Administration website. https://www.fda.gov/media/155049/download. Accessed March 23, 2022.

[19] Molnupiravir emergency use authorization. US Food and Drug Administration website. https://www.fda.gov/media/155053/download. Accessed March 23, 2022.

[20] Hammond J , Leister-Tebbe H , Gardner A , et al. Oral nirmatrelvir for high-risk, nonhospitalized adults with COVID-19. N Engl J Med 2022;386:1397–1408. 35172054 10.1056/NEJMoa2118542 PMC8908851

[21] Jayk Bernal A , Gomes da Silva MM , Musungaie DB , et al. Molnupiravir for oral treatment of COVID-19 in nonhospitalized patients. N Engl J Med 2022;386:509–520. 34914868 10.1056/NEJMoa2116044 PMC8693688

[22] Fact sheet: Biden administration launches nationwide test-to-treat initiative ensuring rapid “on the spot” access to lifesaving COVID treatments. US Health and Human Services website. https://www.hhs.gov/about/news/2022/03/08/fact-sheet-biden-administration-launches-nationwide-test-treat-initiative-ensuring-rapid-on-spot-access-lifesaving-covid-treatments.html. Accessed March 23, 2022.

[23] About CVS health. CVS website. https://www.cvshealth.com/about-cvs-health/our-company-at-a-glance. Accessed March 23, 2022.

[24] Walgreens frequently asked questions. Walgreens website. https://www.walgreens.com/topic/pharmacy/healthcare-clinic/frequently-asked-questions.jsp. Accessed March 23, 2022.

[25] Expanding access to COVID-19 therapeutics. HHS PREP Act Declaration: Ninth Amendment. US Public Health Emergency website. https://www.phe.gov/Preparedness/legal/prepact/Pages/PREPact-NinethAmendment.aspx. Accessed March 23, 2022.

[26] Fact sheet: the Biden administration to begin distributing at-home, rapid COVID-19 tests to Americans for free. The White House website. https://www.whitehouse.gov/briefing-room/statements-releases/2022/01/14/fact-sheet-the-biden-administration-to-begin-distributing-at-home-rapid-covid-19-tests-to-americans-for-free/. Accessed March 27, 2022.

[27] Klepser DG , Klepser ME , Smith JK , et al. Utilization of influenza and streptococcal pharyngitis point-of-care testing in the community pharmacy practice setting. Res Social Adm Pharm 2018;14:356–359. 28479019 10.1016/j.sapharm.2017.04.012

[28] Klepser DG , Klepser ME , Murry JS , Borden H , Olsen KM. Evaluation of a community pharmacy-based influenza and group A streptococcal pharyngitis disease management program using polymerase chain reaction point-of-care testing. J Am Pharm Assoc 2019;59:872–879. 10.1016/j.japh.2019.07.011 31474527

[29] Zhao A, Dangerfield II DT, Nunn A , et al. Pharmacy-based interventions to increase use of HIV pre-exposure prophylaxis in the United States: a scoping review. AIDS Behav 2022;26:1377–1392. 34669062 10.1007/s10461-021-03494-4 PMC8527816

[30] Ritonavir-boosted nirmatrelvir (paxlovid). COVID-19 treatment guidelines. National Health Institutes website. https://www.covid19treatmentguidelines.nih.gov/therapies/antiviral-therapy/ritonavir-boosted-nirmatrelvir--paxlovid-/. Accessed March 27, 2022.

[31] Vogel EA , Billups SJ , Herner SJ , Delate T. Renal drug dosing: effectiveness of outpatient pharmacist-based vs prescriber-based clinical decision support systems. Appl Clin Inform 2016;7:731–744. 27466041 10.4338/ACI-2016-01-RA-0010 PMC5052546

[32] NASPA launches point-of-care testing certificate program. National Association of Student Personnel Administrators website. https://naspa.us/2021/03/naspa-launches-point-of-care-testing-certificate-program/. Accessed March 27, 2022.

[33] COVID-19 resources. Society of Infectious Disease Pharmacists website. https://sidp.org/covid19. Accessed March 27, 2022.

[34] ASHP COVID-19 resource center. American Society of Health-System Pharmacists website. www.ashp.org/covid-19. Accessed March 28, 2022.

[35] Pharmacy and Medically Underserved Areas Enhancement Act. American Society of Health-System Pharmacists website. https://www.ashp.org/advocacy-and-issues/provider-status/pharmacy-and-medically-underserved-areas-enhancement-act. Accessed March 27, 2022.

[36] Pharmacy’s top priority: Medicare provider status recognition. American Pharmacists Association website. https://www.pharmacist.com/Advocacy/Issues/Medicare-Provider-Status-Recognition. Accessed March 27, 2022.

[37] Khazanchi R , Evans CT , Marcelin JR. Racism, not race, drives inequity across the COVID-19 continuum. JAMA Network Open 2020;3(9):e2019933. 32975568 10.1001/jamanetworkopen.2020.19933

[38] Wiltz JL FA , Molinari NM , et al. Racial and ethnic disparities in receipt of medications for treatment of COVID-19—United States, March 2020–August 2021. Morb Mortal Wkly Rep 2022;71:96–102. 10.15585/mmwr.mm7103e1 PMC8774154 35051133

